# Antigen-Presenting Cells in Food Tolerance and Allergy

**DOI:** 10.3389/fimmu.2020.616020

**Published:** 2021-01-08

**Authors:** Elise G. Liu, Xiangyun Yin, Anush Swaminathan, Stephanie C. Eisenbarth

**Affiliations:** ^1^ Department of Laboratory Medicine, Yale University School of Medicine, New Haven, CT, United States; ^2^ Department of Immunobiology, Yale University School of Medicine, New Haven, CT, United States; ^3^ Section of Rheumatology, Allergy & Immunology, Yale University School of Medicine, New Haven, CT, United States

**Keywords:** food allergy, dendritic cells, oral tolerance, monocytes, gut, mesenteric lymph node, Peyer’s patches, macrophages

## Abstract

Food allergy now affects 6%–8% of children in the Western world; despite this, we understand little about why certain people become sensitized to food allergens. The dominant form of food allergy is mediated by food-specific immunoglobulin E (IgE) antibodies, which can cause a variety of symptoms, including life-threatening anaphylaxis. A central step in this immune response to food antigens that differentiates tolerance from allergy is the initial priming of T cells by antigen-presenting cells (APCs), primarily different types of dendritic cells (DCs). DCs, along with monocyte and macrophage populations, dictate oral tolerance versus allergy by shaping the T cell and subsequent B cell antibody response. A growing body of literature has shed light on the conditions under which antigen presentation occurs and how different types of T cell responses are induced by different APCs. We will review APC subsets in the gut and discuss mechanisms of APC-induced oral tolerance versus allergy to food identified using mouse models and patient samples.

## Introduction

Food allergy is a growing epidemic in the developed world, with 6%–8% of children and about 2% of the general population affected in the United States (1–4). A small group of foods including peanut, tree nuts, egg, milk, soy, wheat, fish, shellfish, and sesame cause over 90% of food allergies in the United States. For sufferers of food allergy, consuming the target allergen can lead to various body-wide symptoms including hives, swelling, gastrointestinal distress, cardiovascular, and respiratory compromise, and in rare instances, fatal anaphylaxis (5). The standard of care for food allergy treatment is to avoid consuming the allergenic food and to carry emergency medications in case of accidental ingestion (6). Despite advances made in food allergy treatment with oral immunotherapy, a cure is still elusive. Food allergy greatly affects quality of life, so more treatment options are direly needed (7). To identify therapeutic targets and advance research, it is crucial to understand the mechanisms underlying food allergy.

Food allergy is a type 2 immune reaction to dietary antigens that can manifest in several ways depending on the pathophysiological endotype (8); some forms of food allergy are dominated by the type 2 cellular response, whereas others primarily present with symptoms of the humoral type 2 response. This review will cover what is known about the regulation of the cellular and humoral immune reactions to food antigens by the dominant antigen-presenting cells of the immune system, dendritic cells (DCs).

The cellular immune response of type 2 immunity is coordinated by Th2 CD4^+^ T cells, which produce IL-4, -5, and -13 cytokines as well as chemokines and other chemical mediators; a subset of these T cells also make IL-9. The ensuing cellular response includes recruitment and activation of eosinophils, group 2 innate lymphoid cells, (ILC2s) and basophils, as well as changes to the epithelial barrier (9). ILC2s amplify the Th2 response within the gut by producing IL-5 and -13 and quickly react to the production of alarmin cytokines such as IL-25 from the gut epithelium (10). Th2 cells also induce a population of mucosal mast cells that produce IL-4, IL-9, and IL-13 (11), which expands the intestinal mast cell population while suppressing regulatory T (Treg) cell generation, enhancing susceptibility of anaphylaxis to food allergens (12, 13). Tregs are responsible for oral tolerance, the induction of non-responsiveness to gut antigens including food. Many such Tregs are induced within the gut [peripheral Tregs (pTreg)], and we will cover what is known about this important step in avoiding allergic sensitization to food.

The humoral immune response of type 2 immunity is epitomized by IgE, which is driven by two closely related populations of CD4^+^ T follicular helper (Tfh) cells, IL-4-producing Tfh2 and Il-4 and -13 producing Tfh13 cells (14). In contrast to allergic airway inflammation, mast cells are essential for the allergic IgE-mediated form of food allergy (15, 16). Cross-linking of high-affinity IgE on mature mast cell membranes induces release of the chemical mediators of anaphylaxis, the “weep and sweep” response; this eliminates the target of the IgE antibodies but can be life-threatening. There is also ample data that a positive feedback loop ensues from IgE-mediated mast cell activation, resulting in enhanced cellular type 2 immunity to food allergens (13). Other innate immune cells have also been implicated in contributing to anaphylactic responses in both human and mouse studies including basophils, platelets, macrophages, and neutrophils (17).

Mounting both of these adaptive immune responses begins by activating the correct type of antigen-presenting cell (APC). This requires innate immune activation, since in the absence of activating signals, APCs should induce antigen-specific T cell tolerance. Tolerance is the primary response of the gut immune system to food antigens. Antigen can be acquired by APCs in the gut lamina propria (LP) through multiple access points, including goblet cell-associated passages (18, 19), microfold (M) cell sampling in Peyer’s patches (PPs), and gut lumen sampling CX3CR1^+^ macrophages that pass off antigen to migratory DCs (20). These DCs migrate in a CCR7-dependent manner to provide either activating or tolerizing signals to naïve lymphocytes within gut-associated lymphoid tissues (GALT) (21). GALT are located throughout the intestine and include PPs, mesenteric lymph nodes (MLNs) and isolated lymphoid follicles (ILFs). These are unique cellular niches for induction of tolerance but are also sites for T cell priming and B cell activation. It is important to note that many theories on sensitization to food allergens implicate the skin rather than the gut as the relevant site based on clinical and experimental data (22). Therefore, we will also cover what is known about the APC response in the skin to food allergens.

## APC Populations in the Gut

**Figure 1:**
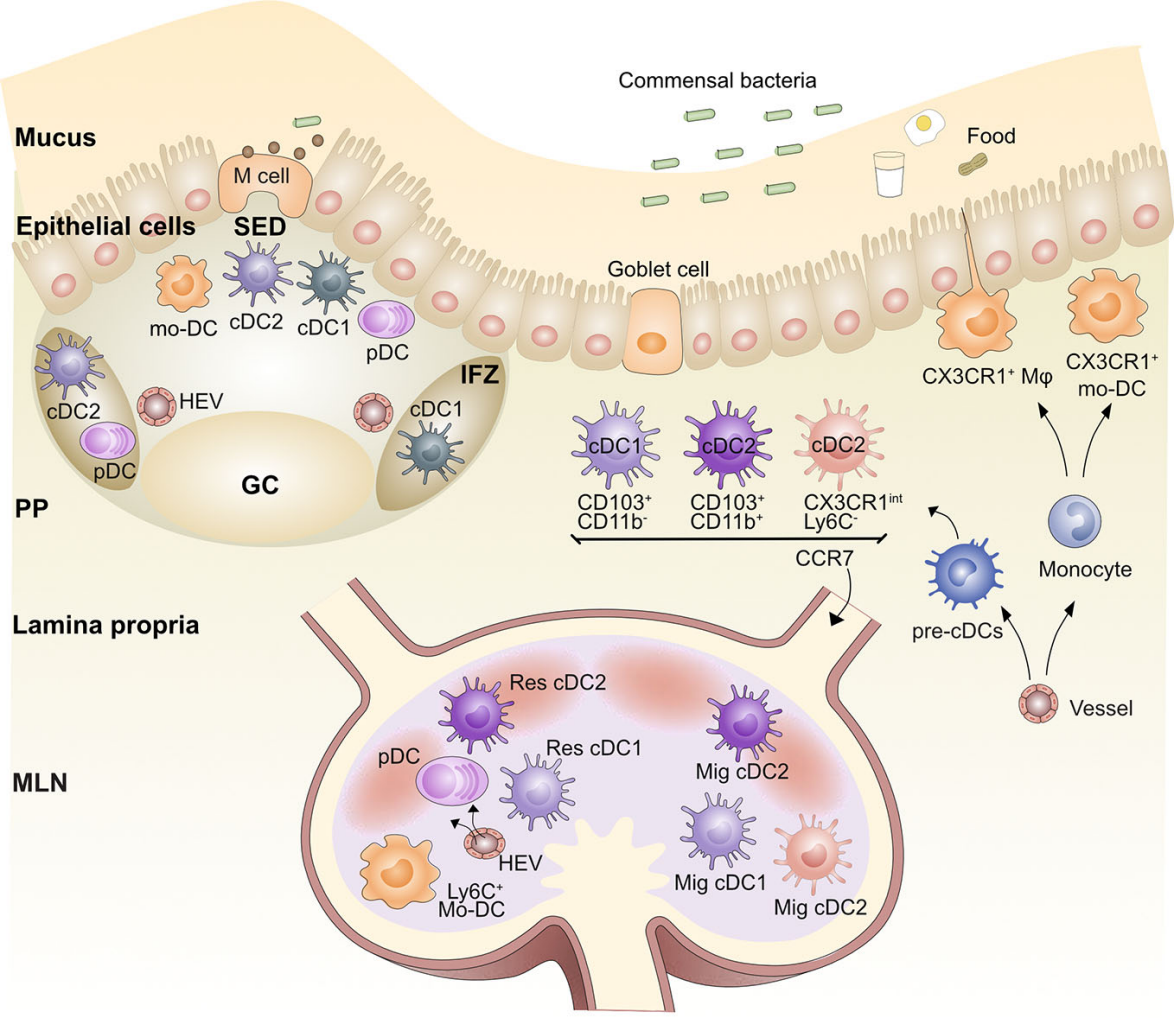
Organization of the gut antigen presenting cell network. Blood pre-cDCs populate the lamina propria (LP), Peyer’s Patches (PP), and mesenteric lymph node (MLN) and differentiate into cDC1s and cDC2s. After being activated by antigen, LP cDC1s and cDC2s are able to migrate *via* afferent lymphatics to the gut-draining MLN *via* CCR7; these DCs are called migratory DCs (Mig DC). Similarly, cDC2s and possibly cDC1s in the subepithelial dome (SED) of the PPs are able to migrate to the intrafollicular zone (IFZ). Lysozyme^+^CX3CR1^+^ monocyte-derived DCs (mo-DC) also populate the SED. Pre-cDCs travel through the blood and seed the MLN and PP, where they differentiate into resident (Res) cDC1 and Res cDC2. Plasmacytoid DCs (pDCs) also populate the LP, PP, and MLN. Blood-derived monocytes differentiate into LP and PP macrophages (Mφ) as well as mo-DCs. Germinal center (GC), Microfold (M) cell, High endothelial venule (HEV).

APCs encompass DCs, monocytes/macrophages and B cells (**Table 1** and **Figure 1**). Little data exist on B cells functioning as APCs in food tolerance or sensitivity; therefore, this review will primarily focus on DCs and monocytes/macrophages in the response to food antigens, starting with a brief introduction on gut APCs.

**Table 1:** Antigen presenting cells in the gut.

	Location	Human	Mouse
cDC1	MLN	Resident: HLA-DR^int^ CD11c^hi^ CD11b^−^ CD8α^+^ XCR1^+^ SIRPα^−^CD141^+^ DNGR1^+^ Migratory: HLA-DR^hi^ CD11c^int^ CD103^+^ CD11b^−^ XCR1^+^ SIRPα^−^CD141^+^ DNGR1^+^	Resident: MHC-II^int^ CD11c^hi^CD11b^−^ CD8α^+^ XCR1^+^ SIRPα^−^ DNGR1^+^ Migratory: MHC-II^hi^ CD11c^int^ CD103^+^ CD11b^−^ XCR1^+^ SIRPα^−^ DNGR1^+^
	LP	CD103^+^ CD11b^−^ XCR1^+^ SIRPα^−^CD141^+^ DNGR1^+^	CD103^+^ CD11b^−^ XCR1^+^ SIRPα^−^ DNGR1^+^
	PP	CD103^+^ CD11b^−^ CD8α^+^ XCR1^+^ SIRPα^−^CD141^+^ DNGR1^+^	CD103^+^ CD11b^−^ CD8α^+^ XCR1^+^ SIRPα^−^ DNGR1^+^
cDC2	MLN	Resident: HLA-DR^int^ CD11c^hi^ CD103^+^ CD11b^+^ XCR1^−^CD1c^+^ SIRPα^+^ CD141^−^ DNGR1^−^ Migratory: HLA-DR^hi^ CD11c^int^ CD103^+^ CD11b^+^ XCR1^−^CD1c^+^ SIRPα^+^ CD141^−^ DNGR1^−^	Resident: MHC-II^int^ CD11c^hi^ CD103^+^ CD11b^+^ XCR1^−^ SIRPα^+^ Migratory: MHC-II^hi^ CD11c^int^ CD103^+^ CD11b^+^ XCR1^−^ SIRPα^+^
	LP	1.CD103^+^ CD11b^+^ XCR1^−^CD1c^+^ SIRPα^+^ CD141^−^ DNGR1^−^ 2. CD103^-^ CD11b^+^ XCR1^−^CD1c^+^ SIRPα^+^ CD141^−^ DNGR1^−^	1.CD103^+^ CD11b^+^ XCR1^−^ SIRPα^+^ 2.CD103^-^ CD11b^+^ XCR1^−^ SIRPα^+^
	PP	HLA-DR^+^ CD11C^+^ CD1c^+^(?) XCR1^-^	CD103^-^ CD11b^+^ XCR1^−^ SIRPα^+^
pDC	MLN, LP, PP	CD11c^−^ CD123^+^ BDCA2^+^(?) BDCA4^+^(?)	CD11c^mid^B220^+^ PDCA1^+^ LY6C^+^CCR9^+^ Siglec-H^+^
Monocyte	MLN, LP, PP	Classical: CCR2^hi^M-CSFR^+^ CD14^hi^CD11b^+^ non-classical monocytes: CCR2^low^ M-CSFR^+^ CD14^low^	Classical: CCR2^hi^M-CSFR^+^ Ly6C^hi^ non-classical monocytes: CCR2^low^ M-CSFR^+^ Ly6C^low^
monocyte-derived cells	MLN, LP, PP	CD14^^+^^ CD11b^+^SIRPα^+^ /CD172^+^BDCA1/CD1c^+^CD226^+^	CD209a^+^ CD11b^+^ CD64^+^CCR2^+^ Ly6C^+^ CD88^+^ SIRPα^+^ /CD172^+^CX3CR1^mid^
Macrophage	MLN, LP, PP	HLA-DR^+^ CD68^+^ CD64^+^ CD209^+^ MerTK^+^ CD14^+^ CD206^+^CD163^+^	CX3CR1^hi ^ CD11b^+^CD64^+^ F4/80^+^MerTK^+^ SIRPα^+^ CD163^+^

Commonly used markers for defining APC subsets in the gut of humans or mice are summarized based on location and subset. “?” indicates that well-accepted markers have not yet been established.

### Dendritic Cell Populations in the Gut

DCs are professional antigen-presenting cells that control both T cell tolerance and priming. Based on ontogeny, phenotype and function, DCs can be divided into conventional/classical DCs (cDCs) and plasmacytoid DCs (pDCs) [for review see (23)]. cDCs are further separated into two subsets, cDC1s and cDC2s (24).

#### Lamina Propria (LP)

Mouse LP is populated by CD103^+^CD11b^-^CLEC9A^+^XCR1^+^ cDC1s, CD103^+^CD11b^+^SIRPα^+^ cDC2s and then a population of cells that are CD103^-^ CD11b^+^DCs (25–29). Human LP have analogous cDC populations with CD103^+^CD141^+^CLEC9A^+^XCR1^+^ cDC1s and CD103^+^CD1c^+^Sirpα^+^ cDC2s (21, 30, 31). Recently, new cDC2 subsets were identified in both human and mouse (32, 33). Since these new DC subsets have not yet been studied in food allergy or tolerance, we will not discuss them. cDC subsets in the LP can migrate into mesenteric lymph nodes (MLNs) *via* CCR7-driven chemotaxis (21, 34, 35). The LP contains a fourth population of CD11b+CX3CR1+ cells; whether these cells migrate to MLNs and prime T cells *in vivo* has been debated (28, 36–39). This is partly due to the mixed origin of CX3CR1^+^ cells in the LP (40). One Ly6C- and cDC-derived subset requires CCR2 for seeding the LP and subsequent CCR7-dependent migration to the MLN (27, 37). In contrast, a Ly6C^+^ monocyte-derived DC (mo-DC) subset, which is also CCR2-dependent, fails to express CCR7 or migrate to MLNs and therefore is not involved in naïve T cell priming in MLN (28, 38, 41, 42). A small population of CD103^-^CD11b^-^ DCs are also present in the LP but are likely cDC1s and cDC2s as they have been shown to either express XCR1 or SIRPα (25). Finally, PDCA1^+^ pDCs responsible for regulating intestinal cDC mobilization towards the MLNs are also present in the LP (21, 43, 44).

#### Mesenteric Lymph Node (MLN)

In the MLN, four populations of CD11c^+^ MHCII^+^ cells are observed using CD11b and CD103 surface staining: 1, cDC1s, which encompass both migratory CD103^+^ CD11b^-^cDC1s from the LP and some CD11b^-^CD8α^+^ resident cDC1s (all are XCR1^+^ and CLEC9A^+^); 2, cDC2, which encompass CD103^+^CD11b^+^ migratory cDC2s and CD11b^+^ resident cDC2s (all are SIRPα^+^); 3, CD11b^+^CD103^-^ cDC2s; and 4, depending on the inflammatory state, a monocyte-derived CD11b^+^CX3CR1^+^ population (25, 27–29). The expression of F4/80, Ly6C, CD64, Zbtb46, and CX3CR1 levels have been used to differentiate populations 3 and 4.

#### Peyer’s Patch (PP)

PP DC subsets have been classically defined in a manner distinct from LP and MLN DCs as CD8α^+^, CD11b^+^, or CD8α^-^CD11b^-^ “double negative” (DN) (45). However, more recent work has united the subsets across a variety of tissues and secondary lymphoid organs (SLOs) using the cDC1 and cDC2 nomenclature (24), including in the gut (25). Using the new classification system, PP DCs fall into two subsets: 1, cDC1s, which includes both CD8α^+^XCR1^+^ and DN XCR1^+^ DCs; and 2, cDC2s, which includes both CD11b^+^ SIRPα^+^ and DN SIRPα^+^ DCs. It is also helpful to maintain the classification of migratory and resident DC subsets in all SLOs, including those without afferent lymphatics like the spleen and PPs, as migration after antigen acquisition occurs between different tissue regions within these sites (23). Resident CD8α^+^XCR1^+^ cDC1s are primarily found in the T cell-rich interfollicular zone (IFZ) of the PP. The heterogeneous populations of DN DCs in PPs have been identified by immunofluorescence staining in the subepithelial dome (SED) and IFZ of the PP (46). With microbial or adjuvant stimulation, SIRPα^+^ cDC2s, including DN DCs and CD11b^+^DCs, can migrate from the SED into adjacent IFZs (47, 48). CLEC9A^+^ cDC1s were noted in the SED of human PPs by immunofluorescence (31). In addition, CD103^+^ cDCs were observed in the SED in rat PPs at steady state but were concentrated in the IFZ after activation (43); these could represent a migratory cDC1 population within the PP, though more work is needed to confirm this. Therefore, we propose to classify PP DC subsets as IFZ-resident cDC1s and cDC2s and SED migratory cDC2s and possibly migratory cDC1s (**Figure 1**). This mirrors the nomenclature in the spleen and LNs. PP PDCA1^+^ pDCs are also found in the SED and IFZ (49). It should be noted that there is little evidence for any of these DC subsets emigrating into PPs from the gut. Therefore, they likely seed the PPs from the blood and then migrate within the PP upon activation.

### Monocytes/Macrophage Populations in the Gut

Monocytes include three main subsets, Ly6C^hi^ (mouse)/CD14^+^(human) classical monocytes and Ly6C^low^ (mouse)/CD14^-^(human) non-classical monocytes and Ly6C^int^(mouse)/CD14^int^(human) intermediate monocytes (50). Classical monocytes express higher CCR2 and require CCR2 for bone marrow egress (51). Monocytes can differentiate into DC-like populations (mo-DCs) or macrophages according to the context, which are difficult to discriminate, and therefore, we will refer to both under the umbrella term, monocyte-derived cells (MCs) (36, 40, 52). A population of CD11c^+^ CD11b^+^ SIRPα^+^ MCs exists in the PP dome that expresses lysozyme and CX3CR1 and can activate T cells *in vitro* (53). A similar population of CD11b^+^CX3CR1^+^ MCs exists in the LP (28, 38–40).

Macrophages in the LP are identified as MHCII^+^F4/80^+^CD11b^+^ CX3CR1^+^ MerTK^+^ in mice (54, 55). MerTK, CD64, CD163, and Sirpα are conserved features of human intestinal macrophages, although at varying levels for macrophage subset (56, 57). Gut macrophages are distinguished from DCs by CD64 expression (38). Although macrophages in most tissues have a dual origin involving both embryonic liver and hematopoietic bone marrow ontogeny, intestinal LP macrophages need continual replenishment from circulating Ly6C^hi^ monocytes in adult mice (38, 39, 58, 59). The function and phenotype of the macrophages that differentiate from these monocyte precursors vary based on the state of inflammation in the gut (40, 54). Although *in vitro* gut macrophages are capable of antigen presentation to naïve T cells, both macrophages and monocytes are rarely observed transporting gut antigens from tissue to LNs to prime naïve T cells, so their primary function is within the gut itself. Inflammatory monocytes enter the LN primarily from the blood rather than migrating from the tissue using CCR2 rather than CCR7 homing signals (60, 61).

## APCs and Oral Tolerance

In the steady state, ingestion of innocuous antigens generally results in oral tolerance. Long-lasting oral tolerance is enforced by Foxp3^+^ pTreg cells induced in MLNs that home to the gut by expressing the integrin α4β7 and the chemokine receptor CCR9 along with T effector cell clonal deletion or anergy (62–65). By raising mice with a diet devoid of dietary antigens, a recent study demonstrated that the majority of small intestinal pTreg cells are induced by dietary food antigens (66). These Tregs suppress CD4^+^ and CD8^+^ T cells, alter mast cell function and re-direct IgE B cell responses (65, 67, 68). Although Tregs can directly promote IgA production through production or activation of TGFβ (69), little is known about the mechanisms or relevance of humoral tolerance in the gut to food antigens. Type 2 inflammation, including IL-4 production from ILC2s, can inhibit the generation and function of these Tregs and can even reprogram them into pathogenic Th2 cells (12, 70), which has been shown in animal models to prevent tolerance and confer a food allergy phenotype.

### Conventional Dendritic Cells

**Figure 2:**
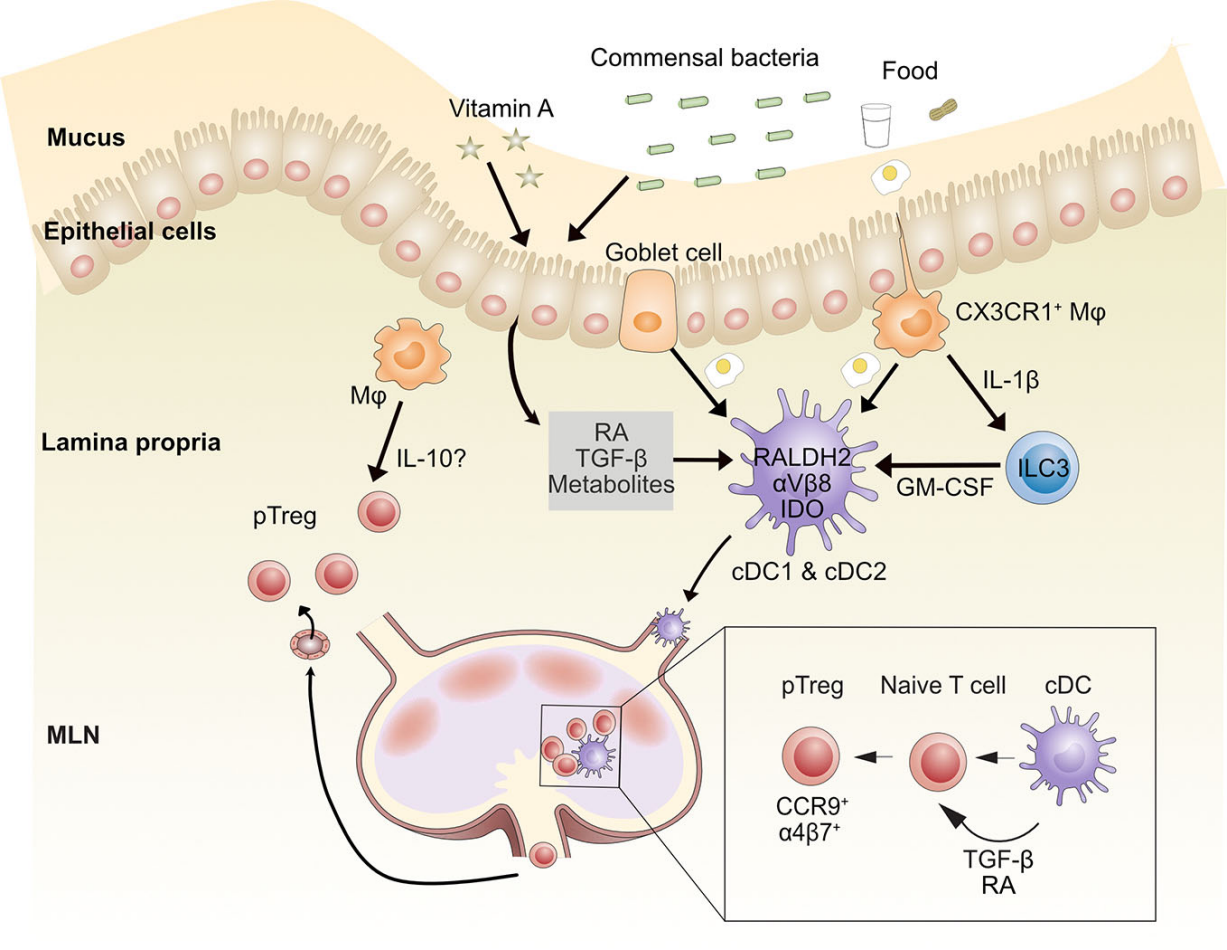
Mechanisms by which gut-associated dendritic cells contribute to oral tolerance. After food ingestion, goblet cells and intestinal resident macrophages sample luminal food antigens and deliver them to LP CD103^+^ cDCs (including CD103^+^CD11b^-^cDC1s and CD103^+^CD11b^+^ cDC2s). Commensal bacterial metabolites, dietary components such as vitamin A, and epithelial cell-derived TGF-β and retinoic acid (RA) imprint tolerogenic properties on cDCs. These cDCs migrate to MLNs through afferent lymphatic vessels and induce naïve CD4^+^ T cells to differentiate into peripheral regulatory T (pTreg) cells through TGF-β and RA. CD103^+^ cDCs induce gut homing molecules CCR9 and α4β7 on pTreg cells, which directs them to recirculate to intestinal tissue. Once in the lamina propria, pTreg cells can be further expanded by macrophages (Mφ), possibly *via* IL-10 production. MLN, mesenteric lymph node; RA, retinoic acid. Conventional dendritic cell (cDC), Innate lymphoid cell type 3 (ILC3).

Intestinal APCs, including cDCs, macrophages and pDCs, play pivotal roles in oral tolerance induction (**Figure 2**). DCs have been implicated in inducing pTreg cell differentiation through multiple mechanisms. After ingestion of foreign dietary antigens, DCs acquire antigen through several routes, including transfer from M cells, macrophages or goblet cell-associated antigen passages but also by sampling the gut lumen using trans-epithelial dendrites (19, 20, 71). However, this latter function may primarily be accomplished by LP CX3CR1^+^ macrophages. MLNs are the primary site of oral tolerance induction (35, 72, 73), although PPs may contribute depending on the nature of the antigen (74). Ablation of cDCs results in the reduction of gut pTreg cells in response to dietary antigen ingestion (75). Gut CD103^+^ cDCs, carrying antigens that are critical for the development of oral tolerance, migrate from the LP to the MLNs in a CCR7-dependent manner (20, 28, 35, 41). Unlike other sites, both cDC1s and cDC2s express CD103 in the gut, and therefore in many studies it is difficult to know which cDC subset is responsible for tolerance. More recent work has distinguished the two cDC populations and found that although murine CD103^+^CD11b^-^ cDC1s are more efficient pTreg cell inducers compared with CD103^+^CD11b^+^ cDC2s, the two subsets may play redundant roles in gut pTreg cell induction and oral tolerance (75, 76).

Several mechanisms have been identified for pTreg cell induction by cDCs. In the intestine, CD103^+^ cDCs express the RALDH2 enzyme, which metabolizes vitamin A to retinoic acid (RA) (41, 75, 77, 78). RA induces the expression of gut-homing molecules CCR9 and α4β7 integrin on T cells (62, 79–82). Human intestinal cDC2s express higher RALDH2 and αVβ8 and induce more Treg cells than cDC1s *in vitro* (30, 83). Murine cDC1s and cDC2s also activate latent TGF-β through integrin αVβ8 (20, 75, 82, 84–86). RA synergizes with TGF-β to induce pTreg cell differentiation *in vitro* (78, 79, 87–90) and IgA class switching of B cells in PPs (86). Mice lacking αVβ8 on DCs have reduced Treg cells in colonic tissue (91). Moreover, TGF-β along with RA can increase β8 expression on cDC1s, thereby creating a positive feedback loop and strengthening the regulatory function of cDC1s (92). Human and mouse gut CD103^+^ cDCs also express indoleamine 2,3-dioxygenase (IDO), an enzyme involved in tryptophan catabolism, which can reduce local tryptophan concentrations and produce immunomodulatory tryptophan metabolites. This can induce Foxp3^+^ Treg cell conversion and oral tolerance (93, 94). An earlier study showed that programmed death ligand 1 (PD-L1, B7-H1) and PD-L2 (B7-DC) expressed on MLN DCs were required for the generation of antigen-specific CD4^+^Foxp3^+^ Treg cells (95). A more recent study instead found that CD11b^-^CD103^+^PD-L1^high^ cDC1s induce Treg cells through RA production and/or activation of TGF-β but that expression of PD-L1 or PD-L2 were dispensable (80). It is unclear whether specific culture conditions explain these inconsistencies, so more work needs to be done to clarify the function of PD-L1 and PD-L2 on DCs in Treg cell induction.

Many aspects of the gut microenvironment promote DC induction of Tregs. MUC2, the building block of gut mucus, imprints DCs to deliver tolerogenic signals promoting pTreg cells and oral tolerance (96). Both mouse and human intestinal epithelial cells can also directly promote the differentiation of tolerogenic DCs and *in vitro* generation of Tregs (87, 88). Finally, we will discuss the effect of the microbiome on gut DC function below.

### Plasmacytoid Dendritic Cells

pDCs can also mediate oral tolerance. Infants who are tolerant to peanut ingestion, but possess peanut IgE, a state called sensitized tolerance, display an increased frequency of pDCs in the blood (97). In cholera toxin (CT)-induced peanut sensitization in mice, expansion of DC numbers by Flt3L, in particular pDCs, inhibits allergic manifestations in the intestine (98). Mucosal pDCs promote the induction of antigen-specific pTreg cells through an autocrine loop involving TGF-β; pDC-ablated mice partly reduce pTreg cell generation in the MLNs after OVA feeding (99). After protein or hapten antigen ingestion, pDCs in the liver and MLN delete antigen-specific CD8^+^ T cells and efficiently induce oral tolerance through an unknown mechanism (100, 101).

### Macrophages

Although gut-resident CX3CR1^+^ macrophages do not migrate to MLNs (28, 41), they contribute to pTreg cell generation and oral tolerance by transferring gut lumen antigens to migratory CD103^+^ cDCs, *via* a mechanism that was shown to be Connexin 43-dependent and required membrane transfer (20). LP macrophages can also maintain Foxp3^+^ Treg cells by a mechanism dependent on IL-10 (102). CX3CR1-deficient mice, which have reduced IL-10-producing F4/80^+^CD11b^+^MHC-II^int^ macrophages, have impaired accumulation of FoxP3^+^ Treg cells in the LP and oral tolerance (62). However, in two colitis studies, CX3CR1^+^ macrophage-derived IL-10 was dispensable for maintenance of colonic Tregs; instead, loss of IL-10 receptor expression on the macrophages themselves impaired mucosal homeostasis (103, 104).

## APCs and the Microbiome in Oral Tolerance

The gastrointestinal tract is colonized by large numbers of commensal microbes that contribute to the maintenance of intestinal homeostasis including protection from food allergy (105). Early life colonization is important for suppressing inappropriate IgE induction (106). Both food-allergic infants and mice demonstrate dysbiosis, and restoring particular bacterial classes such as Clostridium species reduced susceptibility to food allergy and was associated with enhanced Tregs (107–109). The microbiome can promote barrier integrity, which can preclude APCs from encountering food antigen in an inflammatory context. Clostridium species have been shown to promote the production of IL-22, which led to decreased systemic absorption of peanut allergens by increasing intestinal barrier integrity *via* the production of antimicrobial peptides and mucus (109). In addition, bacterially produced SCFA can promote inflammasome activation and IL-18 release in colonic epithelial cells, which then help maintain gut homeostasis in a chemically-induced colitis mouse model; a similar mechanism could be at play in food tolerance as well.

There are several mechanisms by which bacteria may act on APCs to protect against food allergy. First, when certain strains of bacteria like Clostridia metabolize dietary fiber in the gut, they produce short chain fatty acids (SCFA), such as butyrate and acetate, which promote the development of Tregs. SCFA bind to the receptors GPR43 and GPR109A to enhance MLN CD103^+^ DC activity by upregulation of RALDH2, which prevents food allergy development in a murine model (110). A study in milk-allergic children found that children fed with extensively hydrolyzed formula and *Lactobacillus rhamnosus* GG supplements were more likely to outgrow their milk allergy in part because of changes in their microbiome that led to more butyrate in the stool (111), suggesting a possible role for SCFA on human DCs. Recently, metabolism of bile acid by the microbiota has also been shown to promote Treg generation. Bacterial bile acid metabolism generates biologically active steroids. One such product, 3β-hydroxydeoxycholic acid (isoDCA), acts on DCs through the farnesoid X receptor to promote Treg formation (112). It is feasible that these bile metabolism products may play a role in food tolerance as well, but that remains to be tested. Finally, microbiota can help regulate the myeloid cell populations within the gut. Mortha and colleagues showed that microbiota promoted the release of GM-CSF by ILC3s by driving macrophage IL-1β production (113). GM-CSF locally enhanced DC and macrophage numbers and their ability to produce regulatory factors like RA, TGF-β, and IL-10; ablation of GM-CSF reduced Treg cell numbers and impaired oral tolerance (113, 114).

## APCs and Tolerance Induction *via* Immunotherapy

Various forms of immunotherapy are being studied for the treatment of food allergy—these include oral, sublingual and epicutaneous applications of low amounts of food allergens. Immunotherapy alters the cellular and humoral arms of allergy, reducing IgE and enhancing IgG4 (in humans) as well as suppressing T cell, mast cell and basophil reactivity to the target allergen. Immunotherapy has been shown to capitalize on many of the tolerogenic pathways of APCs described above. In particular, cDCs and pDCs from the blood have been shown to adopt, at least transiently, a less inflammatory state after immunotherapy and promote Treg properties *in vitro* (115, 116). Successful food allergen immunotherapy is also associated with increased levels of circulating Tregs (116). In murine studies, immunotherapy with allergens induces TGF-β-producing Tregs in draining LNs capable of homing to the gut, suppressing the allergic response to food challenge and redirecting CD4^+^ effector T cell differentiation away from a Th2 phenotype (68, 117). Looking at sites draining sublingual allergen exposure, migratory cDC2s were proposed to be the dominant APC responsible for Treg induction through a mechanism that, *in vitro*, required RA and TGF-β (118).

## APCs in Food Allergy Pathogenesis

The gut immune system must continuously distinguish innocuous dietary antigens and commensal microbes from pathogens. A breakdown of the default oral tolerance to food leads to abnormal immune responses that manifest as diverse pathologies, such as IgE-mediated food allergy, celiac disease, and eosinophilic gastrointestinal disease, among many others (119). In each of these conditions, adaptive immunity is targeted at a food antigen, presumably all *via* presentation on an APC, but through distinct mechanisms. For example, IgE induction is implicated in IgE-mediated food allergy, but not in celiac disease, which is instead a cell-mediated disease initiated by the presentation of modified gluten on APCs (120). Elucidating the conditions under which APCs are activated in each type of adverse food reactions may provide insight into the differing responses. Here we describe what is known about APCs in the pathogenesis of IgE-mediated food allergy.

### Food as an Innate Immune Stimulus for DCs

**Figure 3:**
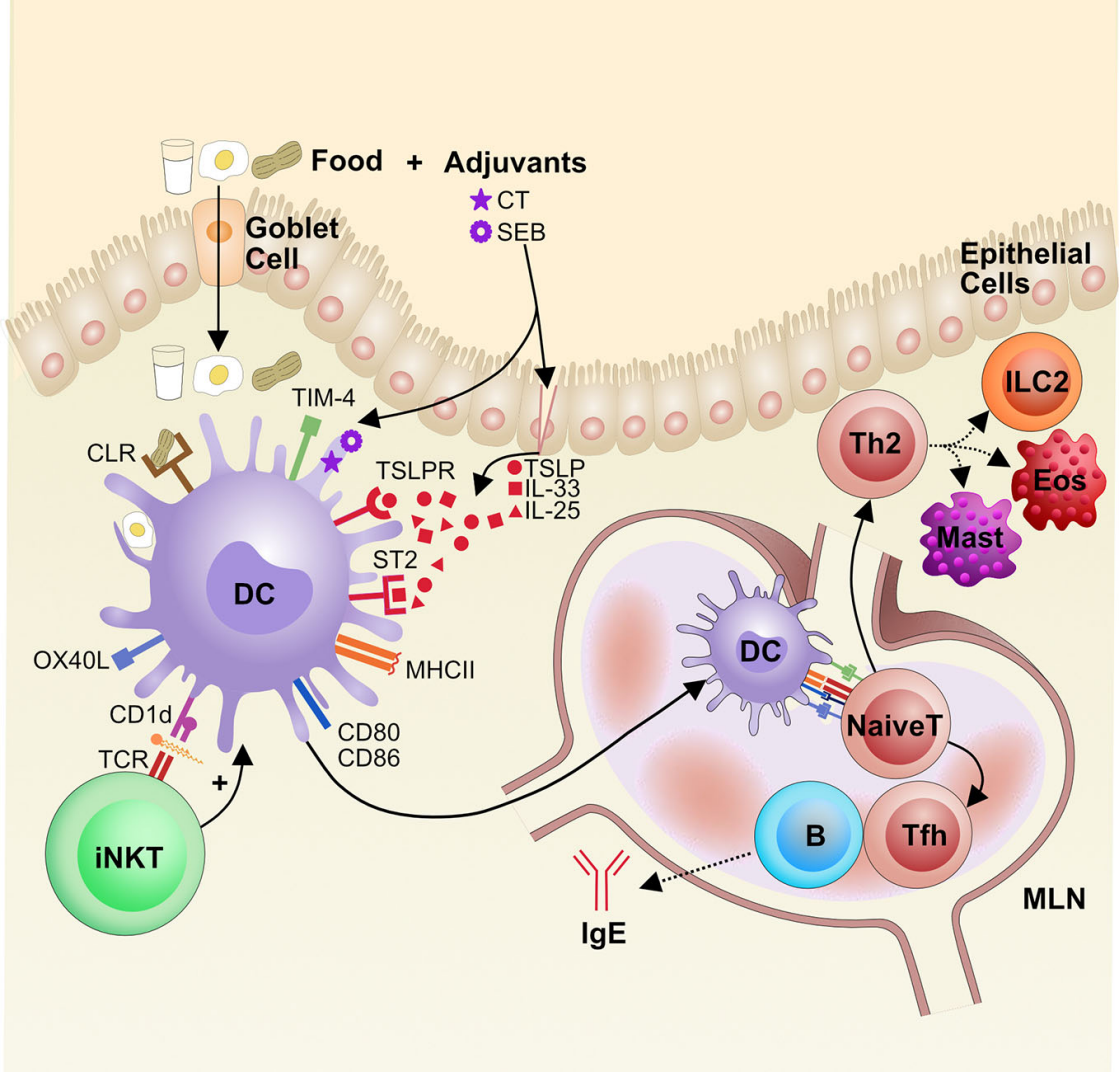
The role of dendritic cells in the pathogenesis of food allergy. Food antigens are taken up from the gut lumen by goblet cells, which shuttle the antigens across the epithelial layer to the LP, where local dendritic cells (DCs) sample the food antigens. If DCs sense innate immune signals, adjuvants that are either extrinsic or intrinsic to the food antigen, they become activated. Some adjuvants damage the epithelial barrier and trigger the release of alarmins, like TSLP and IL-33, that can activate DCs *via* their receptors TSLPR and ST2, respectively. Additionally, food glycoproteins, such as from peanut, can bind to C-type lectin receptors (CLRs) and activate DCs. Lipids from foods can be presented on CD1d to iNKT cells that then reciprocally activate DCs *via* cytokine release. Activation of DCs leads to increased CCR7 for migration to mesenteric lymph nodes (MLN) along with presentation of food antigens on MHCII and increased expression of costimulatory molecules CD80, CD86, OX40L, and TIM-4. Altogether this promotes naïve CD4^+^ T cell priming and differentiation into Th2 cells and T follicular helper (Tfh) cells, which drive cellular and IgE responses in food allergy, respectively. Eos, eosinophils (Eos), Mast cell (Mast), Innate lymphoid cells type 2 (ILC2).

APCs are important in tolerance induction, but they also play a pivotal role in the induction of food allergy (**Figure 3**). Of all the APCs, DCs have the best-defined role in the initiation of food allergy. DCs reside in tissues, where they serve as sentinels that are activated by innate stimuli. The identity of the innate stimuli that can activate DCs to initiate food allergy is unclear, but both intrinsic food components and extrinsic adjuvants are potential innate stimuli currently under investigation.

There is evidence that certain foods can act as auto-adjuvants. Many of these innately immunostimulatory foods are glycoproteins that bind to dendritic cell C-type lectin receptors (CLR), a family of proteins that traditionally bind carbohydrate residues in a calcium-dependent manner (121). One group identified that the glycans on the allergenic peanut protein Ara h 1 bind to the CLR dendritic cell-specific intercellular adhesion molecule-3-grabbing non-integrin (DC-SIGN) on human monocyte-derived DCs and subsequently activate the DCs; these DCs then promote Th2 activation *in vitro* (122). Another group tested the ability of various food allergens and aeroallergens to bind DC-SIGN and the related DC-SIGNR on human monocyte-derived DCs and found that among other allergens, hazelnut, walnut, and egg white could also bind these CLRs. Downstream of these CLRs, the kinases ERK and Raf-1 are upregulated and TNF-α, which is important for DC activation (123), is produced in a partially Raf-1 dependent manner (124).

It has been observed that high-temperature roasting of peanuts increases the allergenicity of peanut proteins (125). Roasting causes peanut protein to undergo the Maillard reaction, which leads to more heat- and digestion-resistant peanut antigens, perhaps allowing for more antigen to reach the relevant sites of IgE induction (126). However, roasting can also lead to the generation of glycoproteins that bind the mannose receptor, a CLR that mediates antigen uptake and appears to play an important role in DC activation. One group has shown that human monocyte-derived DCs take up more roasted peanut protein Ara h 3 than raw Ara h 3, through a mechanism that is partially dependent on the mannose receptor (127). The mannose receptor has also been shown to play a role in peanut protein Ara h 2 uptake by human monocyte-derived DCs *in vitro* (128). Additionally, treating mouse bone marrow-derived DCs with mannose receptor RNAi reduced ovalbumin uptake and DC activation (129). Whether these CLRs are necessary for food allergen sensitization is still unknown.

Food may also act as an intrinsic adjuvant for DC activation by activating invariant natural killer T cells (iNKT). iNKT cells are a population of innate-like cells that display a semi-invariant T-cell receptor that binds lipid antigens presented on the MHC-I-like molecule CD1d on DCs and can in turn promote DC activation (130–132). One group found that sensitization to Brazil nuts is dependent on CD1d lipid presentation to iNKT cells in mice, and human iNKT cells are stimulated by the lipid fraction of Brazil nuts (133). Additionally, lipids from the respiratory allergen *Olea europea* (olive) pollen increase CD1d expression on DCs to activate iNKT cells and also upregulate DC activation marker CD86 (134), a mechanism that could also apply food lipids. Indeed, food lipids from cow’s milk (135, 136), soy (136), and human milk (136) have also been shown to activate human iNKT cells *in vitro*. However, it remains unknown whether iNKT cells orchestrate food allergen sensitization and their exact *in vivo* role in food allergy pathogenesis.

### Gut Adjuvants for Food IgE Sensitization

While the innate immunostimulatory activity of food may bias which foods can act as allergens, it is unlikely to be the only factor affecting the development of food allergy. The innate properties of food cannot explain differential responses to allergens between people, i.e. why most people tolerate food while some develop allergy. Genetic differences can influence some of this susceptibility (137), but the rapidly growing rate of food allergy does not support a solely genetic cause either. Instead, it is likely that there are increasingly prevalent extrinsic factors, such as external adjuvants, that influence activation of DCs to initiate sensitization to food. Accordingly, mouse models have demonstrated that breaking oral tolerance to food antigens, including both cellular and humoral immunity, requires the presence of an adjuvant (138–140). Adjuvants such as cholera toxin (CT) or staphylococcal enterotoxin B (SEB), are most often co-administered orally with food antigens to induce IgE and Th2 cells while inhibiting Tregs. Aluminum hydroxide is another commonly used adjuvant in allergy models that has potent immunostimulatory properties on DCs but is administered in the peritoneum and cannot directly interact with the gut immune system. Therefore, this adjuvant will not be further discussed.

CT is a potent oral adjuvant because it induces both human and mouse DC activation and migration (47, 141–143). CT enters DCs and other cells using the GM1-ganglioside receptor (144, 145). Though the mechanism of action of CT is not completely known, it activates adenylate cyclase, which increases intracellular cyclic adenosine monophosphate (cAMP) levels, which in turn leads to DC activation (146, 147). These CT-activated DCs have been shown to promote Th1, Th2, and Th17 responses (147–149), and they are effective at generating both IgE and IgA in food allergy models. CT is a member of the AB5 toxin family, which includes toxins with similar structures and mechanisms of action such as shigatoxin (*Shigella dysenteriae*), labile toxin (enterotoxigenic *E. coli*), and pertussis toxin. Exposure to other members of the AB5 family may also activate DCs and induce IgE in a similar manner as CT (143, 150). While exposure to CT is an unlikely mechanism of allergy induction in humans, data gleaned using adjuvants can give clues to the broader mechanisms by which innate stimuli initiate IgE responses to food.

SEB is a superantigen made by *Staphylococcus aureus*, which is a common microbial colonizer and determinant of disease severity in people with atopic dermatitis (151). Mouse models of food allergy have used SEB as an adjuvant, both epicutaneously, intragastrically, and intraperitoneally (140, 152, 153). Human monocyte-derived DCs are activated by SEB, at least in part through Toll-like receptor 2 (TLR2), but do not upregulate IL-12 production; accordingly, *in vitro* culture of these DCs with T cells leads to Th2 polarization (154). Using mouse mucosal DCs, SEB was also shown to promote DC activation through the cell surface molecule T-cell immunoglobulin-domain and mucin-domain-4 (TIM-4) and promote T cell activation *in vitro* (153).

Alarmins and damage-associated molecular patterns (DAMPs) are self-molecules that the immune system recognizes as distress signals; they are often released during cell death or damage and are important triggers for DC activation. Uric acid is a DAMP that can activate pattern recognition receptors and thereby initiate adaptive immunity in multiple immunization models; it has also been implicated as an adjuvant for food IgE production (155). Similarly, eosinophil peroxidase released by activated eosinophils activates DCs, which migrate to the MLNs and promote the induction of peanut IgE after immunization with peanut and CT (156). Many studies have focused on cytokine alarmins as part of the innate immune response that initiates food allergy. IL-25, IL-33, and thymic stromal lymphopoietin (TSLP) are cytokines released by damaged epithelium and promote type 2 responses across most tissues (157–159). The role of these cytokines in gut IgE induction with peanut and CT was examined, and IL-33, in particular, was found to be necessary for IgE production, whereas IL-25 and TSLP were dispensable. Mechanistically, this was proposed to work through upregulation of OX40L on activated DCs (160). In a different model using egg-derived ovalbumin and medium-chain triglycerides, dietary lipids that stimulate the release of alarmins from the intestinal epithelium (161), IL-25, IL-33, and TSLP were each necessary for the development of allergy (162). In another model of ovalbumin-directed food allergy, IL-25 activated ILC2s in the gut to produce IL-5 and -13 and, in concert with activated Th2 cells, promoted anaphylaxis (10). As will be discussed later in this section, IL-33 and TSLP have also been implicated in food sensitization through the skin. Therefore, alarmins can trigger type 2 immunity, but whether one alarmin has a dominant role in the initiation of food allergy likely depends on the nature of the antigen, adjuvant and route of exposure.

### Gut DC Populations Involved in Food Allergy

Activated DCs are sufficient to induce food IgE, as evidenced by a study showing that adoptive transfer of splenic and Peyer’s patch DCs from mice sensitized with milk and CT led to milk IgE production in naïve mice (163). Because different DC populations have different functions, it is plausible that food allergens or adjuvants activate a common DC subset that is efficient at priming the requisite T cell populations for IgE responses. Several groups have used mouse models to examine particular sub-populations of DCs activated in food allergy. One group reported that mice sensitized orally with peanut and CT experienced global changes to DC populations in the gut. CD11b^+^ cDCs were increased, and CD103^+^ cDCs were decreased in Peyer’s patches and among intraepithelial lymphocytes and lamina propria lymphocytes; both populations of cDCs were increased in the MLNs, which may represent a net migration of CD103^+^ DC to the MLN (98). Another group also demonstrated that mice orally sensitized with ovalbumin and CT had increased total DC numbers in the MLN. Among these MLN DCs, the CD103^+^CD11b^-^CD8^-^ population was selectively increased (164). These findings were corroborated by a study showing that CD103^+^ MLN DCs activate and migrate in an eosinophil-dependent manner after oral peanut and CT immunization (156). These data suggest that a migratory cDC population in the MLN is important for gut IgE induction when using CT as an adjuvant. However, the exact nature of the DC subsets essential for sensitization remains unclear; specifically, whether DC subsets are redundant for sensitization or operate differently depending on the nature of the allergen and adjuvant is unknown.

### Mechanisms of DC Induction of Food Allergy in the Gut

DCs have been shown to use multiple pathways to induce IgE sensitization to food antigens. First, OX40 ligand (OX40L) on DCs has an important role in Th2 sensitization to food. OX40L is a costimulatory molecule present on activated DCs and important in Th2 development (165). In a mouse model of oral ovalbumin and CT sensitization, activated DCs expressed increased levels of OX40L mRNA, while blocking OX40L with an anti-OX40L antibody in an *in vitro* DC-T cell co-culture reduced type 2 cytokine production (164). Another group showed that OX40L is upregulated after intragastric immunization with peanut and CT in an IL-33 dependent manner and that blocking OX40L *in vivo* in mice reduced peanut IgE and IgG1 levels post-immunization (160).

Another important DC pathway for priming allergic responses involves TIM-4, which is expressed on DCs and binds TIM-1 on T cells to influence Th2 cell development (153, 166). When treated with SEB, primary human DCs upregulate TIM-4 and can drive Th2 differentiation *in vitro* (167). Immunization with peanut and CT similarly led to increased TIM-4 expression that was necessary for peanut IgE production in mice (168). Another group investigated the stimuli for TIM-4 production and found that mast cell tryptase stimulates human intestinal epithelial cells to make galectin-9, a carbohydrate-binding lectin protein. Galectin-9 binds to TIM-3 on DCs and is associated with the production of TIM-4, which is needed for sustaining ovalbumin IgE levels after immunization (169, 170). Increased expression of TIM-4 on DCs may also be mediated by STAT6 and p300 (171).

There has also been interest in the Notch pathway in food allergy. Signaling through Notch receptors on CD4^+^ T cells is important for T cell differentiation; different ligands promote different T cell fates (172). In particular, the Notch ligands Jagged 1 and Jagged 2 are expressed on DCs and promote Th2 differentiation (172, 173). Of note, treatment with CT increases Jagged2 expression on DCs (173), which is consistent with the increased Jagged 2 mRNA seen after ovalbumin and CT immunization in mice (164). Even though Jagged2 expression on DCs is needed for Th2 differentiation *in vitro*, it appears to be dispensable *in vivo* (174).

### DCs in Food Allergy Induction Through the Skin

Systemic IgE can be induced through antigen exposure at sites where the body interfaces with the environment, including the gut, respiratory tract, and skin (175). In particular, defects in the skin barrier are associated with the development of food allergy (22). While there is evidence pointing to the skin as an important site of food IgE induction, the APC subsets and mechanism of action underlying cutaneous sensitization remain only partially understood.

As with gut models of food allergy, adjuvants are used in cutaneous models of food sensitization in mice. CT and SEB have been used topically to induce food IgE (175, 176). Additionally, skin damage leading to alarmin (IL-33, TSLP, and IL-25) release can act as an innate stimulus for DC activation in mouse models of cutaneous allergy; this may mirror the skin barrier break down in people with eczema, who are more susceptible to food allergy (177). There are various methods of incurring or mimicking damage to mouse skin, including by mechanical tape stripping, application of large doses of vitamin D analogs, treatment with proteases, or by directly administering TSLP. These methods have all been used as adjuvants with cutaneous application of food to induce food IgE (139, 178, 179).

In murine models of food allergy, IgE can be induced epicutaneously without extrinsic adjuvants (180–182), differing from most gut sensitization models. In an external adjuvant-free model of peanut allergy, application of peanut extract to depilated mouse skin was able to induce peanut IgE. Peanut extract and Ara h 2 extract had intrinsic adjuvant activity and were capable of initiating IgE to a co-administered milk antigen, alpha-lactalbumin. In response to peanut extract, mouse skin cells made IL-33, which presumably binds to the IL-33 receptor, ST2, on DCs; indeed, the subsequent production of type 2 cytokines in this model was dependent on ST2 signaling (180). The mechanism of innate sensing of peanut leading to IL-33 production by keratinocytes is unclear, but perhaps the ability of glycoproteins on peanut to bind CLRs plays a role, as described in the gut. It is possible that peanut auto-adjuvanticity observed in the skin is stronger than in the gut because the immunostimulatory portion of peanut is sensitive to digestion.

IL-33 also plays an important role in adjuvanted models of cutaneous food sensitization because it can act on DCs and promote food sensitization. In an intradermal ovalbumin and TSLP model of atopic dermatitis and food sensitization, keratinocyte derived IL-33 was necessary for ovalbumin IgE production (183). Similarly, in a model of skin sensitization with peanut and tape stripping to disrupt the skin barrier, IL-33 is increased and contributes to the allergic phenotype (184). The allergic responses from IL-33 in these models likely depend on DC expression of ST2, as in the adjuvant-free epicutaneous model and in other models of IL-33 activated DCs (185, 186).

TSLP, a keratinocyte cytokine that is important in atopic dermatitis pathogenesis, also plays a key role in sensitization through the skin (187). In a mouse model of intradermal ovalbumin sensitization using TSLP as an adjuvant, TSLP signaling in DCs was required for ovalbumin IgE production and subsequent anaphylaxis (188). This response may be mediated by TSLP-induced upregulation of OX40L, which promotes type 2 responses both *in vitro* using human DCs and in mice *in vivo* (189, 190). However, in a mouse tape stripping model of skin injury, while TSLP signaling on DCs was needed for Th2 differentiation, OX40L was not upregulated in skin DCs; however, the Th2 inhibitory cytokine IL-12 was suppressed in skin DCs (191). The different data for the role of OX40L in Th2 differentiation may be due to use of different species and models of investigation between studies. In another mouse model of epicutaneous sensitization using ovalbumin with the vitamin D analog MC903 to induce a skin barrier defect, TSLP-induced basophils were necessary for the development of gut allergy (192). *In vitro*, these TSLP-induced basophils interacted with DCs to increase OX40L expression, which in turn, increased IL-4 production by basophils (178).

The cell types and mechanisms of DC initiation of epicutaneous skin allergy have also been examined. In a tape stripping mouse model of allergic inflammation, skin injury led to DC activation and migration, as evidenced by a population of CCR7^+^MHCII^+^ DCs was found in the skin draining LNs 24 h after tape stripping. These DCs were able to prime T cells to produce type 2 cytokines *in vitro* (191). Similarly, in another model of epicutaneous sensitization, after the application of milk protein alpha-lactalbumin (ALA) and CT, Langerin-negative skin DCs increased expression of MHCII and migrated to skin draining LNs, where they promoted type 2 cytokine production and systemic ALA IgE production (175). In a model of subcutaneous ovalbumin and papain skin sensitization, this migratory skin DC population was also required for ovalbumin IgE production and was found to be PDL2^+^ and dependent on the transcription factor IRF4 (193). It is clear that sensitization through the skin induces a systemic T cell response, that can home to the gut to orchestrate a food-specific response. But skin-derived cues can have gut-specific effects as well. Recent work showed that keratinocyte-derived IL-33 induced by skin damage communicates with cells in the gut to promote IL-25 production and ILC2 activation; this enhanced mast cell numbers and anaphylaxis following oral antigen challenge (194). Therefore, it is clear that a unique skin-gut axis exists that can promote food allergy through numerous mechanisms.

### The Role of Monocytes in Food Allergy

Monocytes have also been implicated in food allergy. Infants who develop food allergy were found to have a higher number of cord blood monocytes at birth; when treated with the TLR4 agonist lipopolysaccharide (LPS) *in vitro*, the CD14^+^ monocytes from allergic children produced more inflammatory cytokines IL-1β, IL-6, and TNF-α that promoted the development of a Th2-like population at the expense of a tolerogenic Treg population (195). Another study similarly revealed that the peripheral blood mononuclear cells (PBMCs) of 1-year-old infants that ended up with persistent egg allergy had more monocytes and DCs that made more inflammatory cytokines upon *in vitro* stimulation than those from children who outgrew their egg allergy (196). It is possible that these blood monocytes are precursors for macrophages or monocyte-derived DCs that participate in antigen presentation of food antigens (197). Altogether, these studies suggest that monocytes are biased to respond differently to inflammatory stimuli in those with food allergy; whether this is a cause of or caused by the food allergic state is unclear. There is also little mechanistic data from animal studies implicating monocytes in IgE sensitization. Future studies would be beneficial for a deeper understanding of the topic.

### The Role of Macrophages in Food Allergy

Contrary to their well-established role in food tolerance, the role of macrophages in food IgE priming is not well understood, and there is scant literature on the topic. Macrophages express DC-SIGN (198) and TIM-4 (199), both of which may participate in the priming of food-specific IgE. Additionally, macrophages are found in tissues throughout the body, including the skin and gut (200, 201), so they are poised to potentially play a role in food antigen presentation. Macrophages that are found in Th2 conditions appear to play an IL-33 dependent role in allergic asthma (202–205). However, given the importance of macrophages within tissues both for tissue homeostasis and presenting antigen to primed effector T cells, rather than as APCs for naïve T cells, it is likely that macrophages will be required for different phases of food allergy pathogenesis than DCs. Therefore, more information is needed to elucidate the exact function of macrophages in food sensitization.

## Conclusion

While much work has been done to examine the role of APCs in priming food IgE, there are still many unanswered questions. In particular, the specific population of APCs that lead to food IgE production in the skin and gut should be identified, ideally using several adjuvants to home in on common mechanisms of food IgE production. It would also be useful to study the APC requirements for other nonpathogenic antibody isotypes to food such as IgA and IgG4 to better understand what APC conditions separate tolerance from allergy. Another fundamental question is the identity of innate immune stimuli that lead to DC activation in human food allergy; an understanding of what natural skin or gut adjuvants lead to food IgE induction would be a significant advance in understanding pathophysiology and potential treatments for food allergy. Additionally, the relevance of monocytes and macrophages to food allergy induction needs clarification. Research on these and many other questions in food allergy are revealing new, unexpected pathways unique to the gut immune system and suggesting exciting new approaches for the diagnosis, prevention and treatment of food allergy.

## Author Contributions

EL, XY, AS, and SE wrote the manuscript and designed the figures. All authors contributed to the article and approved the submitted version.

## Funding

This study was supported by Food Allergy Research & Education (FARE)- The Ira & Diana Riklis Family Research Award in Food Allergy (SE), a gift from the Colton Foundation (SE), R01 AI136942 (SE), the Sean N Parker Center for Allergy and Asthma Research (SE), 5T32AR007107 (EL), and NCATS Grant UL1TR001863 (EL). Yale University provides funds for open access publication in Frontiers.

## Conflict of Interest

The authors declare that the research was conducted in the absence of any commercial or financial relationships that could be construed as a potential conflict of interest.
